# Esophageal Neuroendocrine Carcinoma: A Case Report and Literature Review

**DOI:** 10.7759/cureus.23607

**Published:** 2022-03-29

**Authors:** Vlad Vayzband, Sotirios Doukas, Paola Esparragoza

**Affiliations:** 1 Internal Medicine, Saint Peter’s University Hospital, New Brunswick, USA; 2 Gastroenterology and Hepatology, Saint Peter’s University Hospital, New Brunswick, USA

**Keywords:** total neoadjuvant treatment, esophago-gastro-duodenoscopy, progressive dysphagia, large cell neuroendocrine, neuroendocrine carcinoma of esophagus

## Abstract

An 85-year-old woman presented to the hospital with a five-month history of dysphagia, productive cough, dyspnea, new-onset orthopnea, and weight loss. Thoracic CT revealed a sizeable ulcerative mass within the cervical esophagus with complete luminal obstruction. Esophagogastroduodenoscopy with biopsy demonstrated large neoplastic cells with distant nucleoli. The patient was diagnosed with poorly differentiated large cell neuroendocrine carcinoma and was treated palliatively with esophageal stenting and radio and chemotherapy.

## Introduction

Neuroendocrine tumors are most commonly found within the gastrointestinal tract with the highest prevalence being in the stomach [1]. Much less commonly, esophageal involvement occurs, leading to a diagnostic conundrum due to the nonspecific symptoms and the lack of obvious risk factors. Symptoms are often secondary to the tumor's mass effect, manifesting through its compression of nearby anatomical structures. With incidence rates steadily increasing, esophageal neuroendocrine carcinomas become an important diagnostic differential to consider.

Here, we report this rare neuroendocrine carcinoma case in a patient presenting with chronic obstructive symptoms with progressive dysphagia.

## Case presentation

The patient is an 85-year-old female with a past medical history of chronic obstructive pulmonary disease (COPD), asthma, and gastroesophageal reflux disease (GERD), who presented to the hospital for worsening shortness of breath, difficulty swallowing, coughing, and weight loss. Symptoms started five months ago with a white mucoid-producing cough and exertional dyspnea not alleviated with rest. Due to the chronicity and similarities of the current cough to her usual allergies, she did not seek medical attention until two months later. A subsequent pneumonia diagnosis was made and treated with Levaquin and oral methylprednisolone. Despite the resolution of cough following treatment, new-onset orthopnea and an unintentional 36-pound weight loss over the course of two months occurred. The patient endorsed having early satiety, a reduced appetite, and dysphagia significant for food regurgitation. The review of symptoms was negative for nausea, vomiting, epigastric pain, and stool character changes.

The patient has no family history of cancer and has never had an esophagogastroduodenoscopy (EGD) or colonoscopy. Risk factors included a 30-year history of secondhand smoke from a family member who smoked four packs per day around her. The patient did not recently travel nor had any occupational exposures to allergens.

On presentation, the patient was afebrile, respiratory rate of 22 breaths per minute, oxygen saturation of 96% room air, blood pressure of 142/82 mmHg, and heart rate of 120 beats per minute. Physical examination was insignificant except for a cachectic body habitus with significant muscular wasting and mild biphasic wheezing over the main bronchi area.

**Figure 1:**
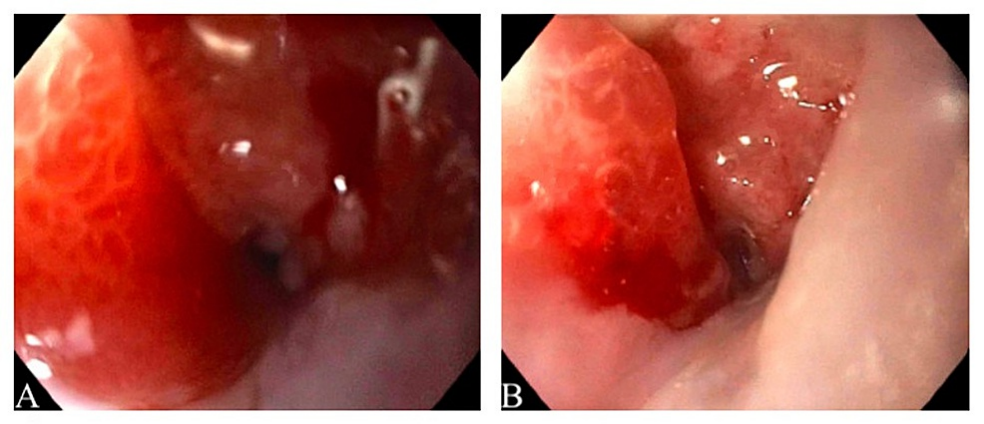
Endoscopic findings: Large ulcerative mass of the upper one-third of the esophagus with complete luminal obstruction.

Thoracic CT revealed a large ulcerating mid-esophageal mass spanning at least 76mm in craniocaudal dimensions, located 20cm from the incisors causing displacement of the bilateral main-stem bronchi anteriorly, with complete bronchial obstruction (Figure 1), and accompanied unilateral mediastinal lymphadenopathy. Given the location and the mass's size, esophagogastroduodenoscopy with biopsy and subsequent placement of an esophageal stent was performed to relieve the esophageal obstruction.

**Figure 2:**
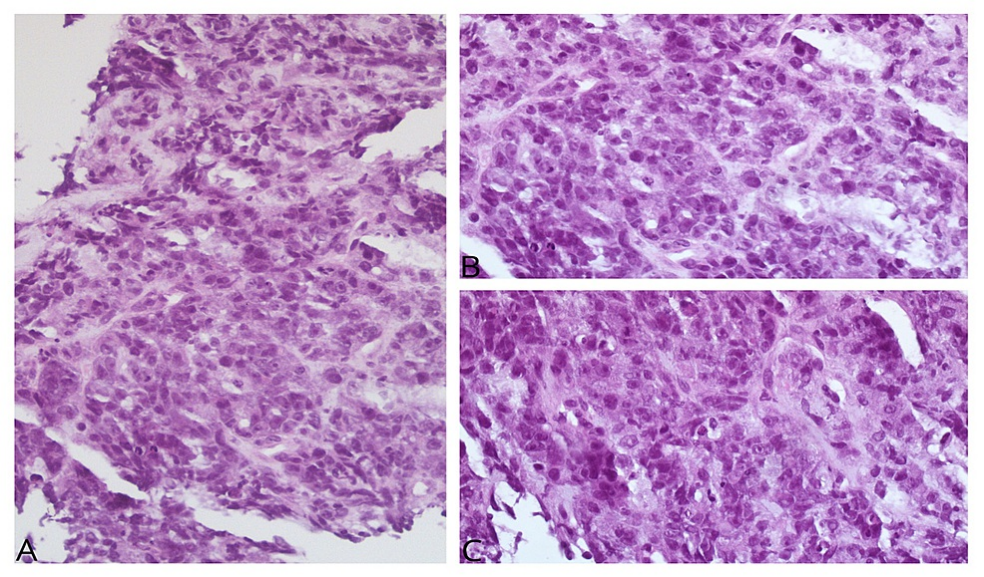
Hematoxylin and eosin stain: Large neoplastic cells with distinct nucleoli under 20x magnification (A) and 40x magnification (B,C)

**Figure 3:**
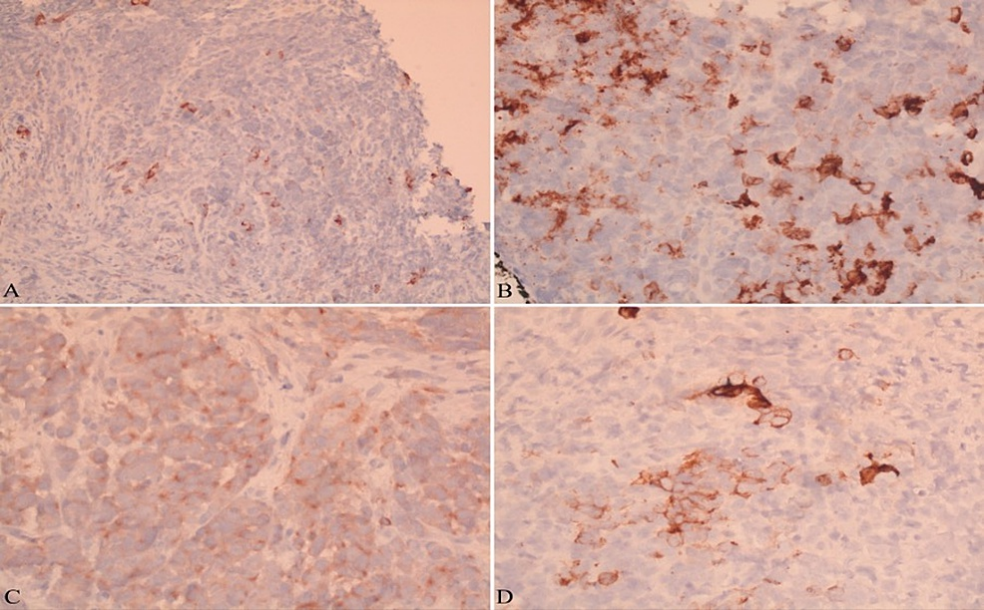
Immunohistochemistry: The tumor was found to be negative for CAM5 (A), and positive for CD56 (B), synaptophysin (C), and pan keratin (D)

The biopsy revealed synaptophysin and CD56 immunostaining positive tumor, indicating poorly differentiated large cell neuroendocrine carcinoma (Figure 2) with associated genetic mutations in MutL homolog 1 (MLH1), MutS homolog 2 (MSH2/6), and PMS1 homolog 2 (PMS2) (Figure 3). Tumor staging revealed it to be T4bN2 as surrounding structures were invaded with the presence of regional lymph node metastasis. 

Due to the location of the mass and the natural course of the disease, a multidisciplinary team was counselled to proceed with urgent radiation and chemotherapy for palliative relief. Resection of the mass was not recommended because of its location and extension into surrounding mediastinal structures. After careful discussion with the hematologist and oncologist, it was determined that the tumor was not sensitive to chemotherapy. Palliative care and hospice were offered but the patient requested to be discharged to rehabilitation. Shortly after the patient passed away while at home with family. 

## Discussion

Primary neuroendocrine carcinomas (NEC) localized to the esophagus are considered a rarity, but despite the relatively low prevalence, the incidence rate is steadily increasing, amounting to approximately 2% of all esophageal carcinomas [2]. The anatomical site plays a vital role in its symptomatology as with other expanding lesions. The patient’s tumor was found 20cm from the incisors, aligning itself approximately at the level of the sternal notch (T3/T4) and within the superior mediastinum. With the cervical esophagus being the most posterior structure, anteriorly expanding lesions can further compromise the trachea and the brachiocephalic vessels of the venous and arterial system. Digestive and then respiratory and hematologic manifestations may ensue with enlargement.

Initial upper gastrointestinal endoscopy should be used to assess the intraesophageal expansion and facilitate the biopsy performance providing histological confirmation of the diagnosis. Treatment and palliative management are then directed by tumor staging, initially evaluated by chest and abdomen CT. Surgically, candidates with stage 1 to 3 cancer are managed with surgical resection, with stage 2 to 3 receiving neoadjuvant therapy first. Unqualified or metastatic patients are managed with palliative chemotherapy or radiotherapy, and symptomatic supportive care. This general approach can be undertaken with any expanding neoplastic lesion. However, additional methods unique to neuroendocrine tumors have been investigated to augment the general schematic. Craig et al. showed that patients with early-stage small cell neuroendocrine carcinoma who underwent surgical resection with adjuvant chemotherapy or radiation had increased longevity than those who underwent surgical resection alone [3], suggesting that the initiation of neoadjuvant therapy may be beneficial even before stage 2 disease progression. Recent geared pharmacological studies also show promising results. The CLARINET and PROMID double-blind clinical trial showed that incorporating a somatostatin analog (SSA) yielded up to two years of progression-free survival in patients with a grade 1 or 2 tumor [4,5]. If tumor progression occurred while on SSAs, peptide-receptor radionuclide therapy also increased the progression-free survival period by another 20 months [6]. Although both these approaches did not yield significant overall survival compared to the controls, they did show size control for the primary tumor that might have clinical significance in the case of compressive disease. Recent studies showed that monoclonal antibodies alone and in combination with chemotherapy could also lead to an increase in overall survivability, including grade 3 tumors [7,8].

## Conclusions

Here, we presented a rare case of esophageal NEC. Although these malignancies are associated with high mortality rates, esophageal NEC management is challenging, given that there are no official guidelines. Given the ongoing development of target therapies, extensive research is necessary to determine the utility of surgical and non-surgical approaches for managing esophageal NEC.

## References

[1] Dasari A, Shen C, Halperin D (2017). Trends in the incidence, prevalence, and survival outcomes in patients with neuroendocrine tumors in the United States. JAMA Oncol.

[2] Kuriry H, Swied AM (2015). Large-cell neuroendocrine carcinoma of the esophagus: a case from Saudi Arabia. Case Rep Gastroenterol.

[3] Craig SR, Carey FA, Walker WS (1995). Primary small-cell cancer of the esophagus. J Thorac Cardiovasc Surg.

[4] Caplin ME, Pavel M, Ćwikła JB (2014). Lanreotide in metastatic enteropancreatic neuroendocrine tumors. N Engl J Med.

[5] Rinke A, Müller HH, Schade-Brittinger C (2009). Placebo-controlled, double-blind, prospective, randomized study on the effect of octreotide LAR in the control of tumor growth in patients with metastatic neuroendocrine midgut tumors: a report from the PROMID Study Group. J Clin Oncol.

[6] Strosberg J (2018). 177lutetium-dotatate delays decline in quality of life in patients with midgut neuroendocrine tumors. Oncotarget.

[7] Lu M, Zhang P, Zhang Y (2020). Efficacy, safety, and biomarkers of toripalimab in patients with recurrent or metastatic neuroendocrine neoplasms: a multiple-center phase Ib trial. Clin Cancer Res.

[8] Patel SP, Othus M, Chae YK (2020). A phase II basket trial of dual anti-CTLA-4 and anti-PD-1 blockade in rare tumors (DART SWOG 1609) in patients with nonpancreatic neuroendocrine tumors. Clin Cancer Res.

